# Supplementary material for: Age-Dependent Changes in Transcription Factor FOXO Targeting in Female Drosophila
Source: Front Genet. 2019 May 7;10:312. doi: 10.3389/fgene.2019.00312 (PMC6514159; doi:10.3389/fgene.2019.00312)

Figure S1

A

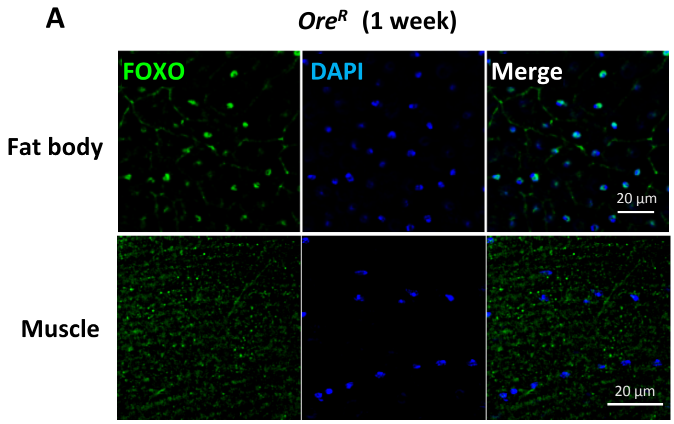

Ore^R (1 week)
FOXO
DAPI
Merge
Fat body
Muscle
20 µm
20 µm

B

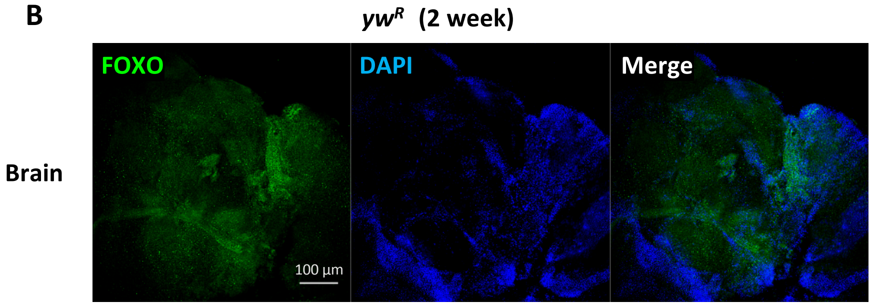

yw^R (2 week)
FOXO
DAPI
Merge
Brain
100 µm

Figure S2

A

Sample	Raw reads	% Alignment
2-week-old ChIP-1	25,554,421	85.74%
2-week-old input-1	40,459,444	96.09%
2-week-old ChIP-2	15,933,943	73.70%
2-week-old input-2	34,196,540	96.26%
5-week-old ChIP-1	15,880,701	92.00%
5-week-old input-1	73,535,040	95.06%
5-week-old ChIP-2	14,515,822	91.76%
5-week-old input-2	40,815,124	95.98%

B

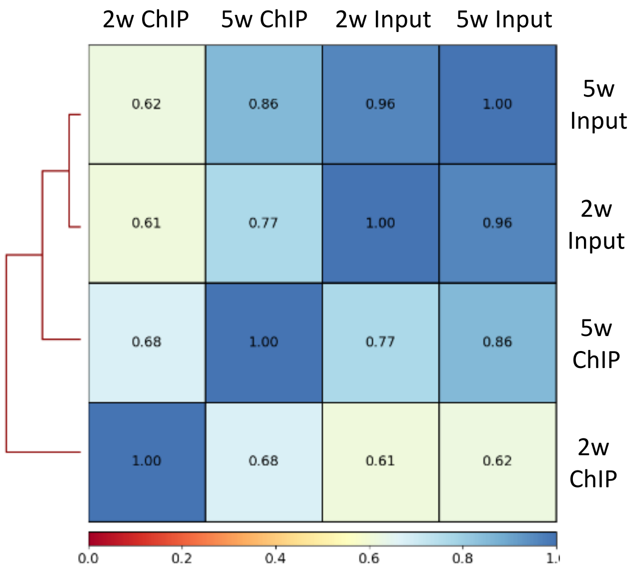

Figure S3

Drosophila Hippo Signaling Pathway

Unique FOXO targets in wild-type
Unique FOXO targets in *chico*$^{-/+}$
Shared FOXO targets

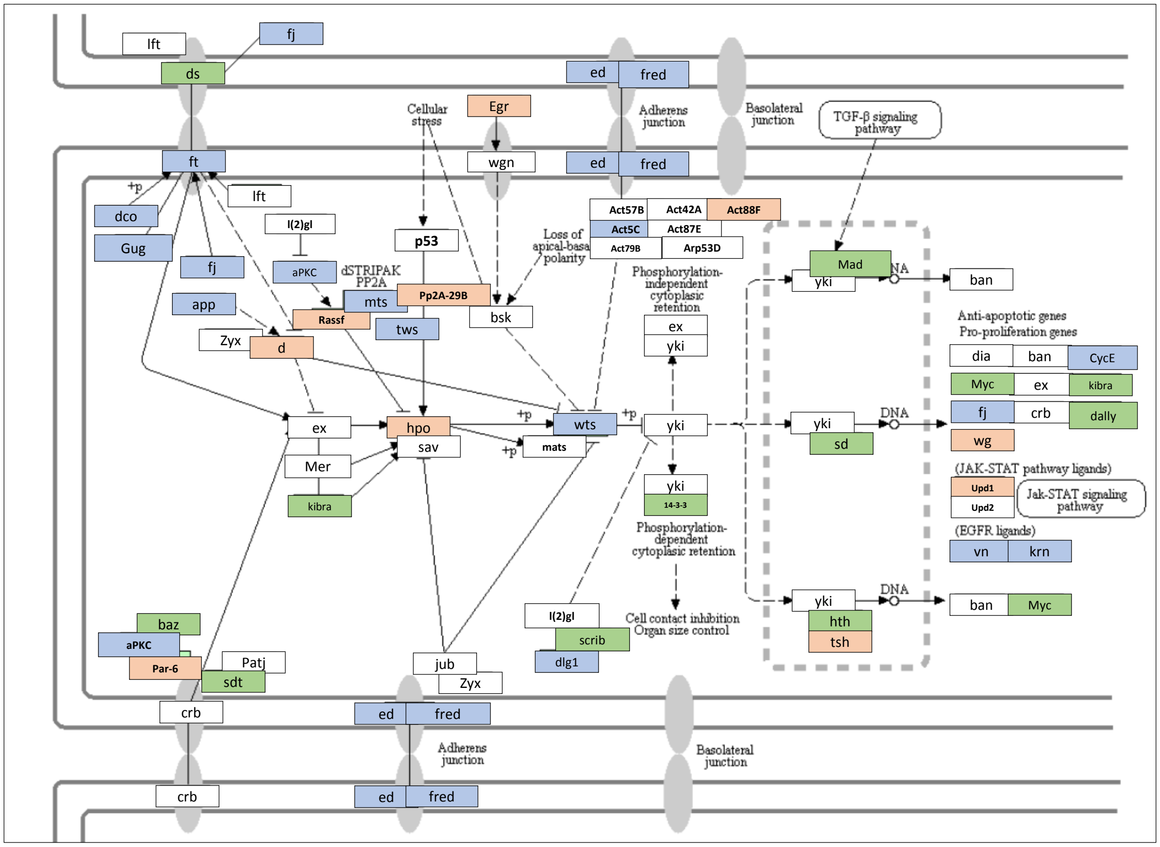

Figure S4

Drosophila MAPK/EGFR Signaling Pathway

Unique FOXO targets in wild-type
Unique FOXO targets in *chico*$^{-/+}$
Shared FOXO targets

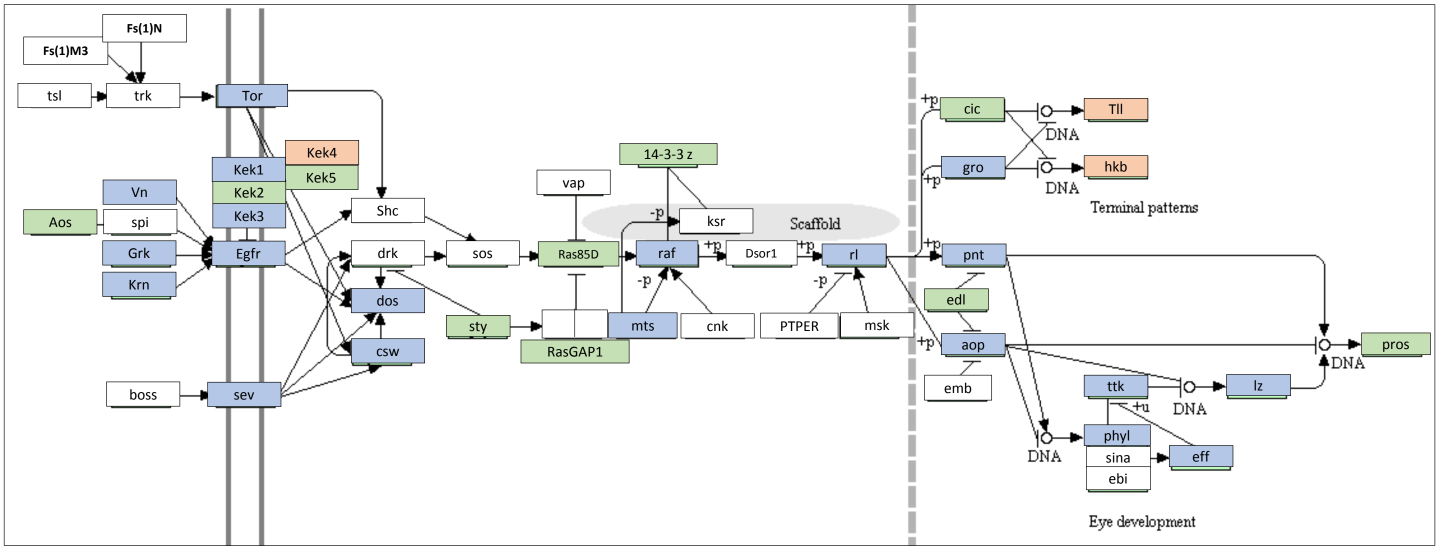

Supplement: FIGURE S1 — (A) Abdominal fat body and flight muscle of female wild-type flies (OreR) stained with anti-FOXO at ages of 2 weeks and 5 weeks. Scale bar: 20 μm. (B) FOXO nuclear localization in adult brain of 2-week-old female ywR flies. Scale bar: 100 μm. [file Data_Sheet_1.PDF]
