# Supplementary material for: Age-Dependent Changes in Transcription Factor FOXO Targeting in Female Drosophila
Source: Front Genet. 2019 May 7;10:312. doi: 10.3389/fgene.2019.00312 (PMC6514159; doi:10.3389/fgene.2019.00312)
Supplement: TABLE S2 — Lists of primers used in qPCR analysis. [file Table_2.pdf]

Table S2

Primer name	Direction	Sequence 5'->3'
Act5C	Forward	TCGCGATTTGACCGACTACCTGAT
	Reverse	TGATGTCACGGACGATTTCACGCT
his1:CG33804	Forward	ACACTTCAAGCAAACTTTGACA
	Reverse	CCAACCTCCTTTGCTCTGAT
his2B:CG33908	Forward	TTCAGGGCTACAACGTTCC
	Reverse	AAACTGAATGCGACCAACATT
InR	Forward	ATAGAACGACGCACTTTCCC
	Reverse	CGCGCGCTCTCCTATTATTTA
bmm	Forward	CACCGCGCCGCAATGAATGTATAA
	Reverse	TTCAATCACTGTTTGTCGGTCGGC
jim	Forward	GAGGCGGGTTTAAGGCTATT
	Reverse	CAGGCAAACAAATCAAAGCAAAC
dlg1	Forward	CTGTTCTCTGTTCTTCTCTTCTT
	Reverse	AGTAGTAGTAGTAGTGGTAGTAGTATAG
